# Macrophage-derived extracellular vesicles: diverse mediators of pathology and therapeutics in multiple diseases

**DOI:** 10.1038/s41419-020-03127-z

**Published:** 2020-10-28

**Authors:** Yizhuo Wang, Meng Zhao, Shuyun Liu, Jun Guo, Yanrong Lu, Jingqiu Cheng, Jingping Liu

**Affiliations:** 1 Key Laboratory of Transplant Engineering and Immunology, Regenerative Medicine Research Center, National Clinical Research Center for Geriatrics, West China Hospital, Sichuan University, Chengdu, China; 2 Institutes for Systems Genetics, Frontiers Science Center for Disease-related Molecular Network, West China Hospital, Sichuan University, Chengdu, China; 3 Department of Critical Care Medicine, West China Hospital, Sichuan University, Chengdu, China

**Keywords:** Mechanisms of disease, Immunotherapy

## Abstract

Macrophages (Mφ) are primary innate immune cells that exhibit diverse functions in response to different pathogens or stimuli, and they are extensively involved in the pathology of various diseases. Extracellular vesicles (EVs) are small vesicles released by live cells. As vital messengers, macrophage-derived EVs (Mφ-EVs) can transfer multiple types of bioactive molecules from macrophages to recipient cells, modulating the biological function of recipient cells. In recent years, Mφ-EVs have emerged as vital mediators not only in the pathology of multiple diseases such as inflammatory diseases, fibrosis and cancers, but also as mediators of beneficial effects in immunoregulation, cancer therapy, infectious defense, and tissue repair. Although many investigations have been performed to explore the diverse functions of Mφ-EVs in disease pathology and intervention, few studies have comprehensively summarized their detailed biological roles as currently understood. In this review, we briefly introduced an overview of macrophage and EV biology, and primarily focusing on current findings and future perspectives with respect to the pathological and therapeutic effects of Mφ-EVs in various diseases.

## Facts

- EVs can carry and transfer various bioactive molecules towards recipient cells and thus participate in the cell-cell communication during disease pathology and tissue regeneration.
- Macrophages play essential roles in the pathology of multiple diseases, and their released EVs are also believed to participate in these courses.
- Mφ-EVs can serve as potential therapeutic targets as well as promising agents for the treatment of diseases due to their similar abilities to macrophages.

## Open questions

- How can we properly regulate the release or the embedded contents of Mφ-EVs to prevent the development of diseases?
- How can we precisely modulate Mφ-EVs to exert diverse functions in response to different microenvironments or disease states?
- Is there any method to generate the reprogrammed/reengineered Mφ-EVs with improved yield and biofunction?

## Introduction

Macrophages are key innate immune cells that circulate in the blood and reside in nearly all tissues with self-renewal capacity and tissue-specific characteristics^1^. Macrophages initially originate from highly heterogeneity hemopoietic progenitors to perform multiple functions that depend on the various stimuli. Macrophages constitute the first barrier against invading pathogens, which are activated and exhibit differential phenotypes in response to a variety of endogenous and exogenous danger signals, mediating the homeostasis of the immune system and multiple tissues. However, abnormal macrophage responses may induce immune disorders and uncontrolled inflammation, which have been implied in many diseases. For example, damage-associated molecular patterns (DAMPs) released after kidney injury activate toll-like receptors (TLRs) and nuclear factor-κB (NF-κB) in macrophages, stimulating reactive oxygen species (ROS) and inflammatory cytokines release that further aggravates inflammation and renal injury^2^. Generally, activated macrophages communicate with various types of target cells to exert their immunomodulatory effects via direct cell-to-cell contact and/or release of the secretome including cytokines and extracellular vesicles (EVs).

EVs are a group of membrane-enclosed vesicles that are naturally released by nearly all types of cells. EVs can be divided into multiple subtypes, such as exosomes, microvesicles, apoptotic bodies, exomeres, and large oncosomes, based on their different origins and sizes. EVs package proteins, nucleic acids, and metabolites of parental cells, and are thought to exhibit similar properties to their parent cells. Recently, EVs have been recognized as vital information carriers that transfer their cargos from parent cells to recipient cells, modulating the physiological or pathological processes in recipient cells. The functions of EVs derived from macrophages in various disease states have been widely investigated, and increasing evidence indicates that these EVs play key roles in the diseases progression. Thus, a comprehensive understanding is needed of macrophage-derived EVs (Mφ-EVs) and their roles in the disease pathology and treatment. In this review, we discussed the important studies with respect to the biological and therapeutic effects of EVs from macrophages in various diseases.

## Macrophage polarization and functions

Macrophages are highly heterogeneous immune cells that act in response to various stimuli. Depending on the microenvironments, macrophages exhibit different phenotypes and can be roughly divided into two subtypes: classically activated macrophages (CAMs, M1-like macrophages) and alternatively activated macrophages (AAMs, M2-like macrophages)^3–5^. With the increasing understanding of macrophages, M2-like macrophages have been further subdivided into the M2a, M2b, M2c, and M2d subtypes based on their gene expression profiles. In general, M1-like macrophages comprise the majority population during early inflammation against danger signals, and then they skew towards M2-like macrophages exhibiting immunoregulatory effects to facilitate tissue repair, regeneration, and fibrosis^6^. The phenotype switch between M1-like and M2-like cells is intimately associated with the disease development. Although the functions of these two types of macrophages are largely distinct, they work collectively to regulate tissue homeostasis.

### M1-like macrophages

**Fig. 1:**
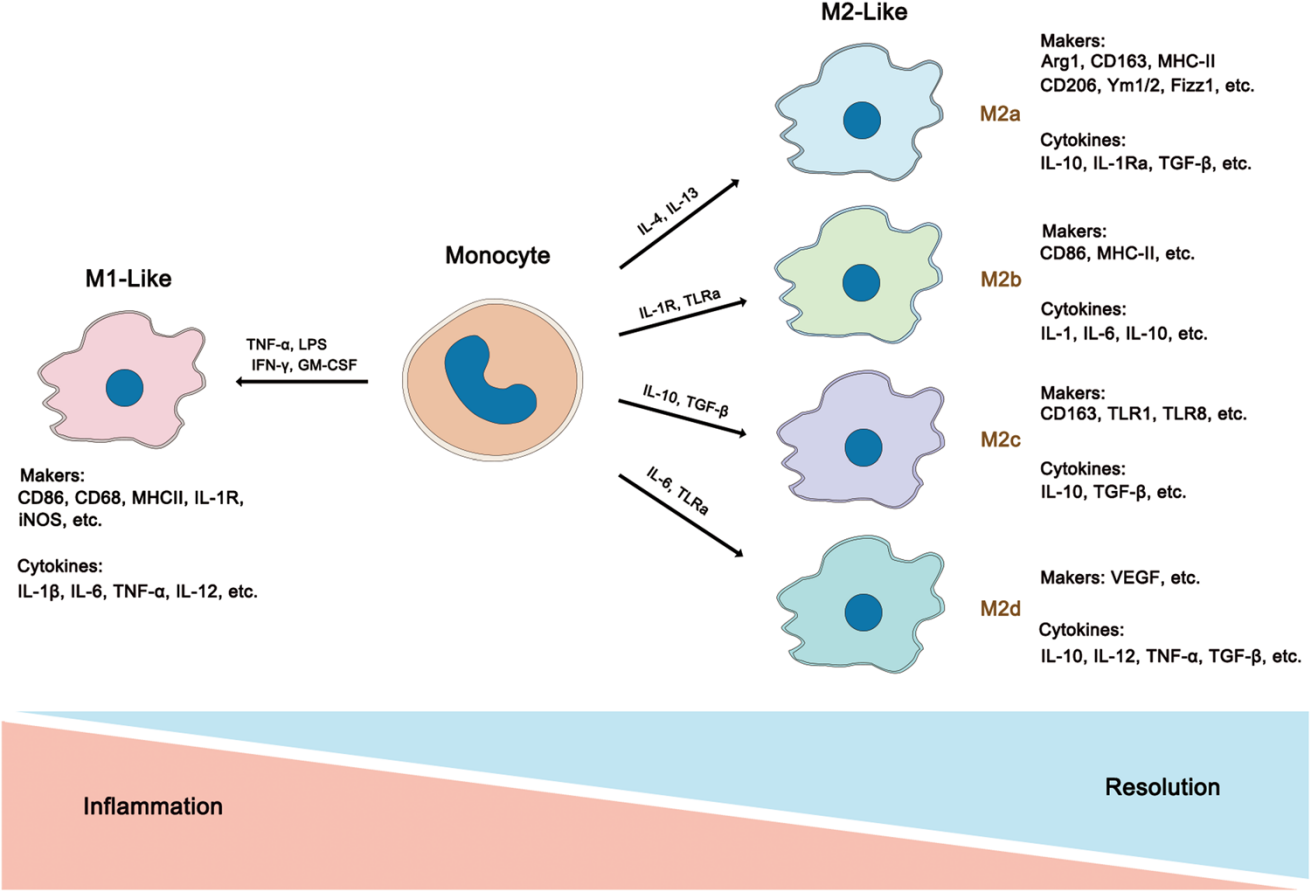
The heterogeneity and characterizations of macrophages. Macrophages could be roughly divided into two subtypes (M1-like and M2-like, while M2-like macrophages can be further differentiated into M2a, M2b, M2c, and M2d phenotypes.) depending on their different microenvironmental stimuli. All of these phenotypes express different cytokines, chemokines, and receptors which give rise to their different functions respectively. Generally, M1-like macrophages mainly induce proinflammatory responses and usually associated with Th1 response while M2-like macrophages contribute trophism and tissue tolerance. Furthermore, M2a is mainly mediating tissue repair and remodeling and Th2 responses; M2b is commonly responsible for immunoregulation; M2c mainly functions in phagocytosis, and M2d participates in angiogenesis in tumor.

M1-like macrophages play important roles during the early stage of pathogen invasion and inflammatory diseases and are typically induced by interferon-γ (IFN-γ), TNF-α, granulocyte monocyte colony-stimulating factor (GM-CSF), or lipopolysaccharide (LPS) in vitro. M1-like macrophages are characterized by several markers, such as CD86, CD68, TNF-α, MHC class II molecules, inducible nitric oxide synthase (iNOS), NOS2, and suppressor of cytokine signaling 3 (SOCS3; Fig. 1). M1-like macrophages secrete high levels of proinflammatory cytokines such as interleukin-1β (IL-1β), IL-6, IL-12, IL-23, and TNF-α, promoting the inflammatory and cytotoxic responses^7^. In addition, M1-like macrophages generate high levels of reactive oxygen species (ROS) and reactive nitrogen species (RNS) to fight against invading pathogens^8^. iNOS acts on l-arginine to produce nitric oxide (NO), which mediates antibacterial and antifungal responses^9^.

When foreign pathogens such as fungi, bacteria and parasites, M1 macrophages release proinflammatory cytokines and induce T helper 1 (Th1) cells differentiation, exhibiting enormous proinflammatory response to deal with these pathogens, which is beneficial for individuals fighting against early stage pathogens invasion. However, excessive proinflammatory and cytotoxic responses can cause severe tissue damage^10^. M1-like macrophages are involved in many inflammatory diseases such as rheumatoid arthritis, hepatitis, inflammatory bowel disease, metabolic syndrome, and diabetes.

### M2-like macrophages

Compared to M1-like macropahges, M2-like macrophages are more likely to contribute trophism, phagocytosis, and induction of tissue tolerance rather than acting as efficient killers. M2-like macrophages are typically induced by T helper 2 (Th2) cytokines (Fig. 1). M2a macrophages are induced by IL-4 and IL-13 via activation of the STAT6 pathway through the common receptor IL-4Rα, which primarily mediates tissue repair and antifungal response^11,12^. Activation of STAT6 can induces the production of arginase-1 (Arg1), which then degrades arginine into polyamines and prolines, thereby promoting cell proliferation and collagen deposition for tissue repair. M2b macrophages are generated upon exposure to immune complexes and IL-1R or TLR agonists and primarily participate in regulating the immune response^13^. M2c macrophages are induced by IL-10, which express the Mer receptor tyrosine kinase and exhibit strong anti-inflammatory and phagocytic effects^14^. M2d macrophages, also called tumor-associated macrophages (TAMs), belong to a newly identified branch of macrophages that are induced by TLR agonists and IL-6^12^. M2-like macrophages are identified by mannose receptor (MR/CD206), Arg1, IL-10, MHC class II molecules, peroxisome proliferator-activated receptor (PPARγ), Fizz1 and YM1/2 family members.

In brief, M2 macrophages primarily participate in tissue repair, immunoregulation, and fibrosis. M2 macrophages often display lower capacity for antigen presentation and oxidants production and higher levels of certain anti-inflammatory factors, such as IL-10 and TGF-β, thereby resolving deleterious inflammatory conditions^15^. However, hyper-activation of M2-like macrophages may induce tissue fibrosis, which is characterized by excessive extracellular matrix (ECM) deposition and destruction of normal tissue structure, resulting in organ dysfunction. In addition, M2-like macrophages also promote tumor progression and metastasis. TAMs are the most abundant immune cell population in tumors^16^, primarily originating from circulating monocytes and providing an immunosuppressive niche that supports tumor invasion. Mechanistically, TAMs secrete matrix metalloproteinases (MMPs) and induce vascularization of tumor tissue by producing growth factors, such as vascular endothelial growth factor (VEGF), platelet derived growth factor (PDGF), and transforming growth factor (TGF)-β^17^. Conversely, depletion or reprogramming of TAMs toward an M1-like phenotype has shown potential for cancer therapy^18^. Although TAMs are believed to be intimately associated with the anti-inflammatory TME, the traditional view of TAMs as skewed M2-like macrophages might be oversimplified. Since tumors are evolving tissues and molecules within TME and vary at different stages, the phenotypes of TAMs are dynamically altered in response to different TMEs^19,20^.

Unlike in vitro conditions, macrophages usually develop mixed phenotypes in vivo in response to disease conditions, and they are difficult to separate using classical M1 or M2 surface markers. For example, it has been reported that macrophage populations display a mixed M1/M2 phenotype in obese patients^21^. The dynamic balance between M1-like and M2-like macrophages tightly controls the disease outcomes, suggesting that regulation of macrophage phenotype is a promising strategy for disease treatment. In addition to cytokines release, increasing evidence indicates that EVs are also critical mediators for intercellular communication between macrophages and target cells or tissues during physiological and pathological processes. In the following sections, we discuss the biological properties of EVs.

## Properties of extracellular vesicles

### Diverse sub-populations of EVs

Depending on their different origins and sizes, EVs were initially categorized into exosomes (Exos, ~30–200 nm), microvesicles (MVs, ~200–1000 nm), and apoptotic bodies (ABs, ~1–5 μm). However, with the increasing appreciations of EVs, many other sub-populations, such as exomeres (<50 nm) and large oncosomes (LOs, ~1–10 μm), have been recently identified^22^. Briefly, ABs originate from the plasma membrane and are often produced when cells undergo apoptosis, exhibiting a broad size range between 1 and 5 μm in diameter. Most ABs are engulfed by phagocytes through receptor recognition by factors such as Annexin V, C3b, and thrombospondin on their surface, which have served as well-accepted markers of apoptotic bodies^23^. Most apoptotic cells are eliminated by phagocytes in the form of ABs, a crucial biological process for avoiding autoimmunity^24^. Exomeres, first identified in 2018, are vesicles smaller than 50 nm lacking external membrane structures, and HSP90 has been proposed as a marker for exomeres. Exomeres contain abundant metabolic enzymes and signature proteins involved in the glycolysis and mTORC1 pathways^25^. However, detailed information and the biological function of exomeres remain largely unknown^26^. LOs are a large sub-populations of EVs (~1–10 μm) that are cancer cell-specific and are derived from the plasma membrane with high expression of ARF6 protein. Secretion of LOs is strongly associated with tumor aggressiveness^27–29^. However, it is unclear how much overlap in the markers and functions exists between LOs and other tumor-derived EV sub-populations.

Despite much progress in the EV field, our understanding of the heterogeneity and diverse function of EV sub-populations is still in its infancy. Currently, the most common method to distinguish EV subtypes is dependent on their size^30^. However, due to a lack of specific markers, identification of any pure EV sub-population is not easy once they have been isolated in vitro. It is now believed that EVs are not only limited to these currently reported sub-populations^22,31^. More detailed and thorough investigations of EV populations are required, which will provide a better understanding of the diverse roles of EVs in disease pathology and treatment. In the literature related to macrophage-derived EVs, some sub-populations, such as exomeres, ABs, and LOs, are rarely involved. Therefore, we primarily focus on the biological roles of macrophage-derived Exos and MVs in the following sections.

### Regulation of EV biogenesis

**Fig. 2:**
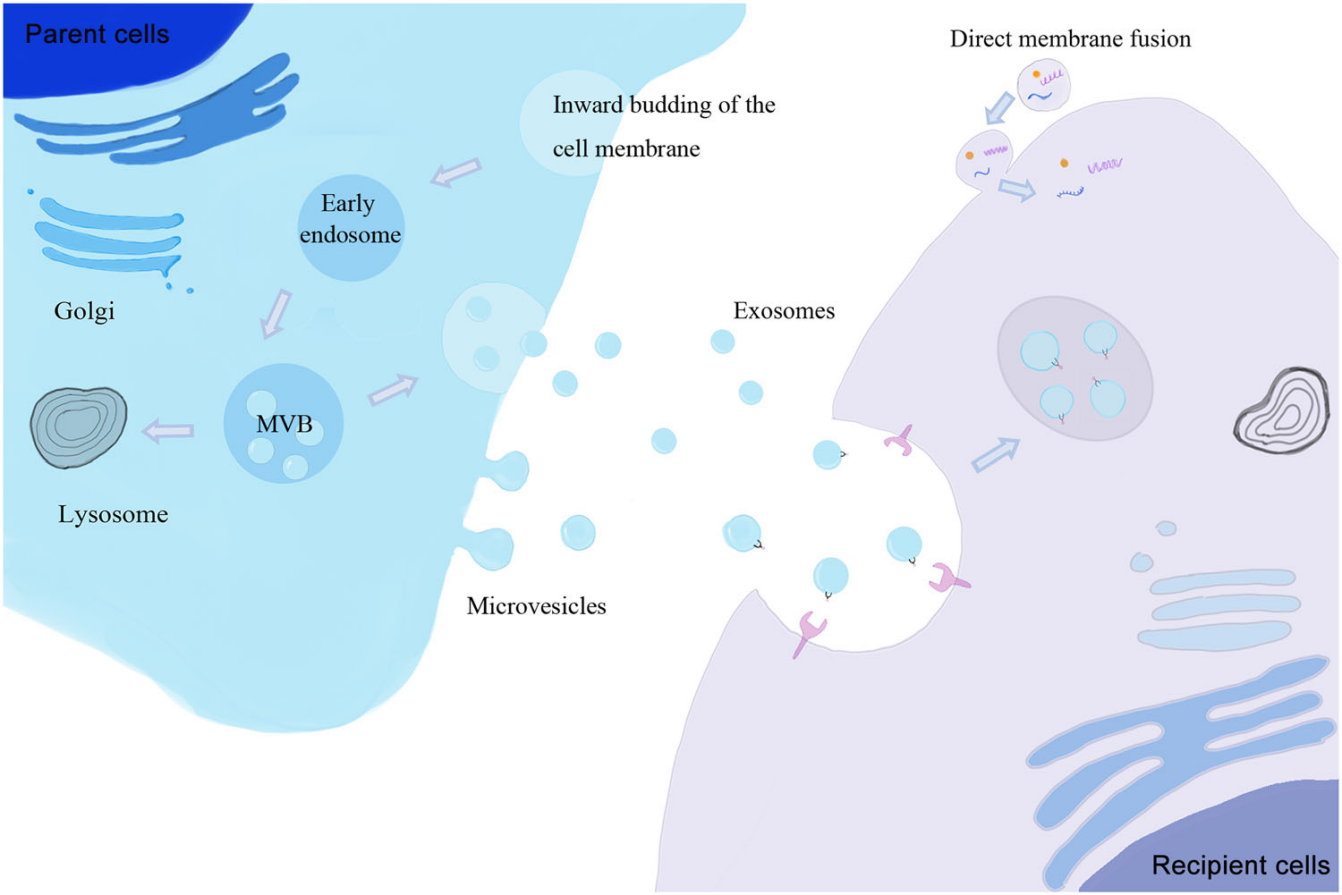
Biogenesis and cellular uptake mechanisms of EVs. There are two major subtypes of EVs known as the Exos and the MVs. Exos are released by the fusion of plasma membrane with MVB which is generated by inward budding of endosome. While MVs are released by direct outward budding of plasma membrane. During cellular uptake, contents within released EVs from parent cells can be transmitted into the cytoplasm of receptor cells by membrane fusion and/or endocytosis. In this endocytosis process, clathrin-mediated endocytosis (CME), macropinocytosis, and phagocytosis are regarded as the most common pathways.

EVs are a group of nano-sized membranous vesicles released by live cells, that were initially considered to comprise a cell waste removal mechanism^32^. However, researchers have now recognized that EVs could carry and transfer biological molecules from parent cells to recipient cells, participating in intracellular communication during disease pathology and tissue regeneration^33^. Based on their different origins and sizes, EVs are further categorized into at least two categories, exosomes (Exos, ~30–200 nm) and microvesicles (MVs, ~200–1000 nm)^34,35^. Briefly, EVs derived from the endosomal pathway are known as Exos and others derived from the plasma membrane are identified as MVs. Exos are primarily released by the fusion of plasma membrane (PM) and multivesicular bodies (MVBs)^36^. Internalization of PM produces early endosomes at the beginning, followed by invagination of endosomes that generates quantities of intraluminal vesicles (ILVs) within the endosomal compartments, leading to the formation of MVBs, which further fuse with the PM to release these ILVs to extracellular spaces, which then become Exos (Fig. 2). Although the detailed mechanism is not fully understand, it has been reported that the endosomal sorting complex required for transport (ESCRT) is intimately associated with the formation of Exos^37,38^. However, knockdown of ESCRT allows the continued formation of Exos, suggesting that other mechanisms in addition to ESCRT are also involved in Exo biogenesis^36,39,40^. One of the alternative pathways involves synthesis of the sphingolipid ceramide^41^, which may have synergistic effects with ESCRT in the biogenesis of Exos. Another ESCRT-independent pathway is the family of tetraspanins, such as CD63, which plays important roles in the regulation of Exo biogenesis^42–45^. Furthermore, Rab proteins, which belong to the Ras-like small GTPase superfamily, have also been found to be associated with Exo release^46,47^.

MVs are thought to primarily originate from directly outward budding of the PM (Fig. 2). Despite their distinct origins, the membrane-trafficking process is involved in the formation of both MVs and Exos. Hence, they may share some common mechanisms of biogenesis. For example, ESCRT and Rab family have been reported to participate in MV formation as well^46,48^. Ras superfamily proteins, such as ADP-ribosylation factor 6 (ARF6), have also been proposed as key regulators of MV biogenesis^49–51^. ARF6 induces PM rearrangement, as well as alteration of actin dynamics and cytoskeleton which are involved in MV formation. In addition, high Ca^2+^ concentration promote MV release via reorganizing of the cytoskeleton^52^. However, the specific process of MV biogenesis remains controversial, and additional studies are required to reveal the exact mechanism of MV biogenesis.

### Cellular uptake mechanism of EVs

EVs can encase multiple types of bioactive molecules such as proteins, RNAs (e.g., mRNAs, miRNAs, mtRNAs, and lncRNAs), DNAs (e.g., mtDNA), and lipids from parent cells. Released EVs are further taken up by recipient cells and regulate gene expression or signaling pathways in these cells. Several mechanisms, such as protein interactions, endocytosis, and direct membrane fusion, have been implicated in the cellular uptake process of EVs (Fig. 2). Protein interactions between EVs and recipient cells may promote subsequent endocytosis. After treatment with Proteinase K to degrade proteins, EVs exhibited a dramatic decrease in cellular uptake rates, suggesting the important roles of protein interactions in this process^53^. In addition, uptake of EVs by endocytosis seems to be the most common pathways, including clathrin-mediated endocytosis (CME), macropinocytosis, and phagocytosis. The cellular uptake of EVs through the CME pathway depends extensively on cholesterol that is enriched in lipid rafts^54^. Flotillins is a common marker protein of lipid rafts that has intimate associations with endocytosis in a CME independent pathway. Another proposed mechanism is the direct membrane fusion of EVs and recipient cells, and several proteins, such as Rab and SNAREs, participate in this process^55^. Moreover, acidic pH conditions, such as in tumors and the stomach, also promote membrane fusion of EVs with target cells^56^. Although several mechanisms have been proposed, it seems that the cellular uptake of EVs involves more than one single mechanism, and multiple pathways work together simultaneously to modulate this process.

### EV isolation and characteristic methods

Isolation of EVs is the first step that directly determines the quality of subsequent research. Currently, several EV isolation methods have been established, and each method has its own advantages and disadvantages. Ultracentrifugation (UC) is the most common isolation method, which obtain EVs based on differential centrifugal force^57^. In brief, liquid samples go through a series of centrifugation to remove cells, debris, apoptotic bodies, and finally collect EVs by UC. UC is widely used in EV isolation due to its high suitability for multifarious liquid samples and simplicity compared to other methods. However, damage to the EVs structure caused by UC is irreversible and may influence subsequent tests. Contamination by aggregate proteins is also a considerable issue^57^. Density gradient flotation is often applied to improve the purity of EVs depending on their density, which is performed through a gradually increasing density to isolate EVs. However, both UC and density gradient flotation are time-confusing and labor-intensive. Other methods including ultrafiltration, size-exclusion chromatography (SEC), immunoaffinity, and precipitation are also applied in EV isolation. Ultrafiltration and SEC are both based on sizes. Although ultrafiltration is time-saving compared to UC, low yield largely impedes its application. EV isolation by SEC is believed to have high purity and integrity compared to other methods. However, elution in SEC may reduce EVs concentration and influence EVs functions^58,59^. Although immunoaffinity exhibits high purity and isolates EVs through specific antibodies that recognize surface markers on EVs, it unsuitable for large volume samples. Recently, several commercial isolation kits have been established that are largely based on the precipitation of EVs in polymer solution. While the high output and convenient process have aroused interest, impurity with lipoproteins and introduced polymers during precipitation has raised some concerns^60^.

Recently, newer EV isolation and detection methods have also been developed. For instance, microfluidic platforms, including size-based separation, immunoaffinity-based separation, and dynamic separation, are primarily based on the size and immunoaffinity of EVs. Despite some advantages of microfluidics, such as high purity, cost-efficiency, and portable properties, shortcomings, such as complicated photolithography fabrication and capturing EVs with only targeted proteins, also exist. Furthermore, nanoscale flow cytometry (nanoFACS) has been used to analyze and sort EV sub-populations using specific antibodies against EV marker proteins^61,62^. Due to its high-resolution, nanoFACS obliterates noise and background and determines the cellular source of EVs, which has made it extremely valuable^63^. Asymmetrical flow field flow fractionation (AF4) is another isolation method that separates EVs based on their different sizes^25,64^. AF4 technology is coupled with a multidetection system composed of ultraviolet-visible spectroscopy (UV) and multiangle light scattering (MALS), which separates and characterizes EVs according to their different diffusion coefficients, since particle size positively correlates with its diffusion coefficient. Although many novel tools have been developed, there is still no current standard for EV isolation or analytic methods, with each method having its own strengths and weaknesses. Therefore, it is necessary to select an appropriate EV isolation method based on the purpose of study.

Characterization of EVs primarily involves morphological and molecular identification. Transmission electron microscopy (TEM) and nanoparticle tracking analysis (NTA) are commonly employed to detect the complete spherical structures and nano-scales of EVs respectively. Western blotting is usually performed to detect EV protein markers, such as TSG101, CD63, ALIX, etc. Notably, none of these methods alone can characterize EVs, and EV characterization requires several methods used collectively^36,65^.

## Pathological roles of Mφ-EVs in disease

**Fig. 3:**
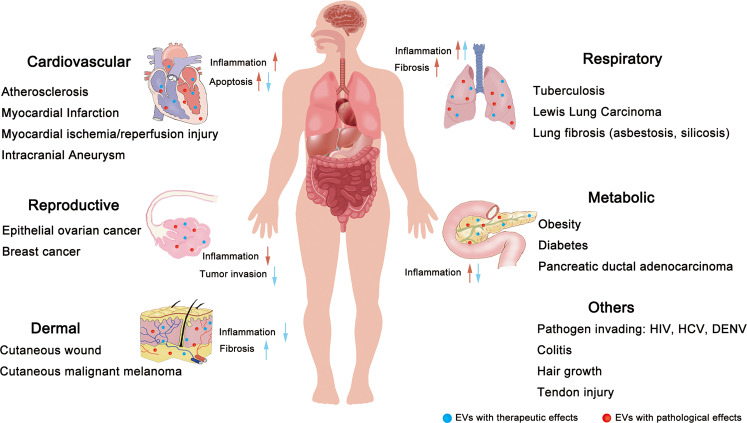
Summary of the current findings for various diseases associated with macrophages-derived EVs. EVs derived from macrophages contain different types of constitutes that can affect the functions of multifarious systems in vivo. Specifically, the red arrows imply for the pathological roles of EVs in diseases and the blue ones imply for their therapeutic roles.

**Fig. 4:**
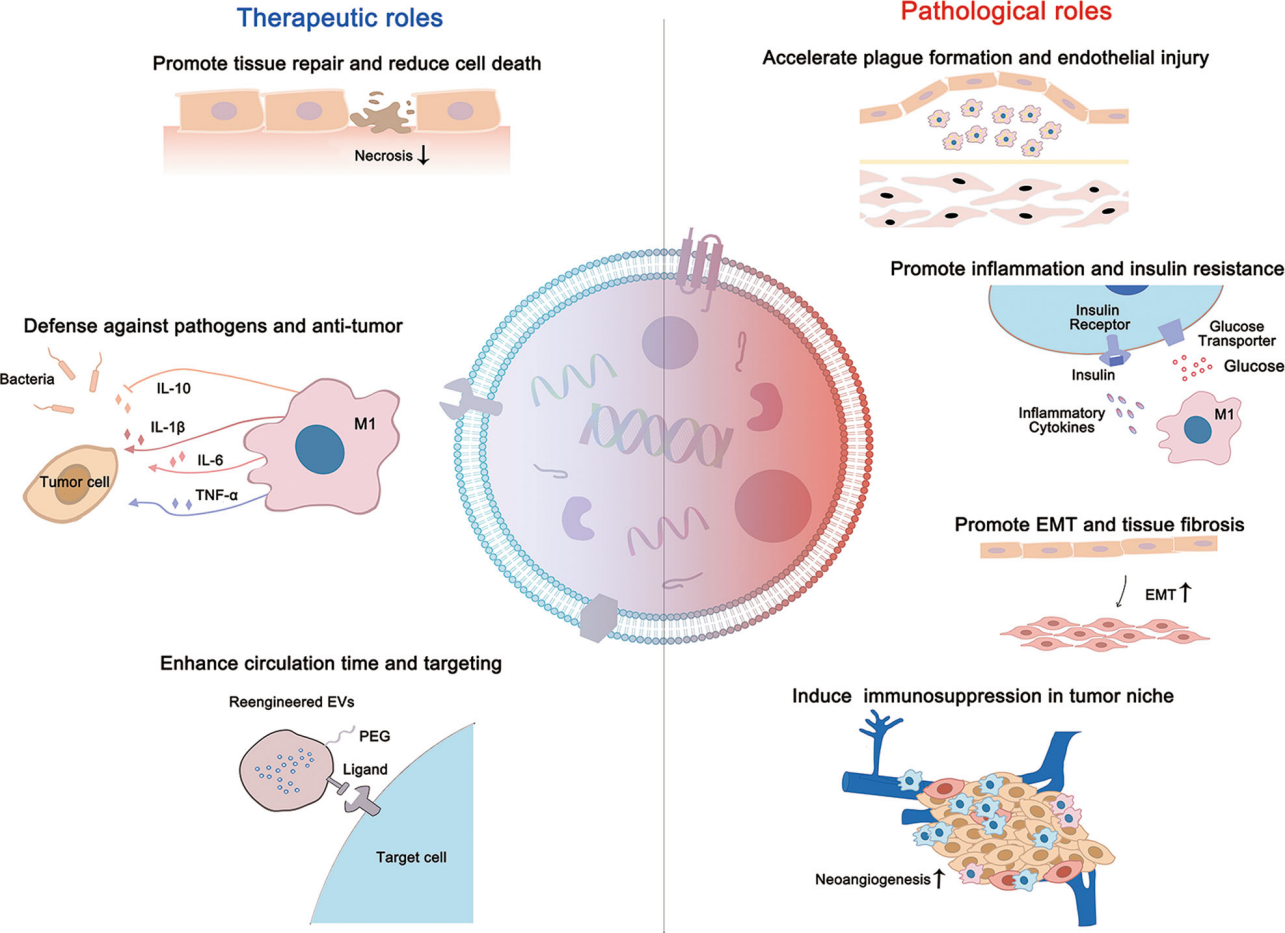
Summary of the diverse roles of Mφ-EVs in multiple diseases. Mφ-EVs can exhibit therapeutic effects to counter pathogens and tumors and promote tissue repair. However, EVs from dysfunctional Mφ can induce excessive inflammatory response, EMT, endothelial injury, and tumor immunosuppression.

Macrophages-derived EVs (Mφ-EVs) deliver abundant proteins, lipids, and genetic information among cells to modify the phenotype and function of target cells. However, contents of Mφ-EVs may vary with different Mφ phenotypes or microenvironments. For example, EVs from polarized and naïve macrophages display distinct miRNA profiles^66^. Since macrophages usually develop complicated and mixed phenotypes in response to different diseases or different phases even in the same disease in vivo, it is not easy to identify the exact subsets (e.g., M1 or M2) of their EVs. Hence, we discuss the roles of macrophages-associated EVs in the pathology of different diseases in the following sections (Table 1 and Figs. 3 and 4).

**Table 1:** Summary of the pathological roles of Mφ-EVs in diseases.

Disease models	EVs source and Isolation methods	EV subtypes (diameter)	Effective molecules	Pathological mechanisms	References
*Cardiovascular*					
Atherosclerosis	- Mouse peritoneal Mφ with oxLDL - Precipitation	EVs (40–300 nm)	miR-146a	Decreased migration of naïve macrophages by downregulation of IGF2BP1 and HuR target to β-actin	^70^
	- THP-1 cell line with oxLDL - Precipitation	Exos (30–130 nm)	Not studied	Decreased the growth and tube formation abilities in ECs	^71^
	- THP-1 cell line with oxLDL - Ultracentrifugation	Exos (undefined)	lncRNA GAS5	Elevated apoptosis of ECs with increasing expression of expressions of P53, Caspase 3, Caspase 7, and Caspase 9	^72^
	- J774a.1 cell line with oxLDL - Ultracentrifugation	EVs (tens to hundreds nm)	Integrins	Enhanced migration and adhesion abilities of VSMC by activating ERK and Akt pathway	^74^
	- RAW264.7 cell line with nicotin - Ultracentrifugation and density gradient fractionation	Exos (30–100 nm)	miR-21-3p	Enhanced migration and proliferation in VSMC by inhibiting PTEN pathway	^75^
Myocardial infarction	- Mouse BMDM with Ang II - Ultracentrifugation	Exos (40–100 nm)	miR-155	- Decreased proliferation of cardiac fibroblast by downregulating Ras and ERK expression - Increased inflammatory response in cardiac fibroblast by activating STAT3 pathway	^78^
Hypertension	- THP-1 cell line with Ang II - Ultracentrifugation	Exos (undefined)	miR-17	Increased inflammation in ECs by upregulating adhesion molecules ICAM-1 and PAI-1	^79^
Intracranial aneurysm	- IA patients’ PBMCs with GM-CSF - Ultracentrifugation	Exos (30–120 nm)	miR-155-5p	Enhanced proliferation and migration of VSMCs by downregulating GERM1	^80^
*Metabolic*					
Diabetes	- Mouse ATM from obesity adipose tissue - Ultracentrifugation	Exos (30–120 nm)	miR-29a	Impaired insulin sensitivity by inhibiting PPARγ pathway	^86^
	–	Exos (30–100 nm)	miR-155		^87^
	- THP-1 cell line with LPS - Precipitation	Exos (undefined)	Not studied	Elevated expression of relevant inflammation gene in adipocytes such as CXCL5, SOD, C3, and CD34	^88^
Premature	- Mouse BMDM from *Ercc1* knockout mice - Ultracentrifugation	Exos (30–80 nm)	*Ercc1*^*−*^	- Elevated glucose uptake by increasing GLUT-1 expression in PPCs - Elevated inflammatory response by activating NF-κB in PPCs	^89^
*Infectious*					
Tuberculosis	- RAW 264.7 cell line with *M.tb* - Sucrose gradient centrifugation	Exos (50–100 nm)	Not studied	- Elevated inflammatory response in BMM via transmigration of mycobacterial components - Accelerated the formation of granuloma by recruiting and activating Mφ towards lung tissue	^92^
Acquired Immune Deficiency Syndrome	- Human PMDM with HIV-Bal - Iodixanol Gradient	Exos (undefined)[75] MVs (~300 nm) and Exos (~60 nm)[76]	HIV-1 viral RNA	Containing abundant viral constituents to uninfected MDM	^93,94^
	- Human PMDM with HIV-Bal - Ultracentrifugation			Enhanced HIV-1 infection by increasing proinflammatory cytokines release	
*Tumor*					
Ovarian cancer	- THP-1 cell line and human peripheral blood mononuclear cells derived Mφ with M-CSF and IL-4 - Precipitation	Exos (undefined)	miR-21-5p and miR-29a-3p	Elevated Treg/T17 ratio in CD4^+^ T cell by suppressing STAT3 pathway to promote tumor growth	^97^
	- THP-1 cell line with IL-4 - Precipitation	Exos (50–150 nm)	miR-223	Promoted drug resistance by activating PTEN-PI3K/AKT pathways in epithelial ovarian cancer cells	^99^
Pancreatic ductal adenocarcinoma	- THP-1 cell line with IL-4 - Precipitation	Exos (~90 nm)	miR-501-3p	Enhanced the migration and invasion of PDAC cells by activating TGF-β pathway leading to tumor formation and metastasis	^98^
*Fibrosis*					
Tendon injury	- Mouse BMDM - Ultracentrifugation	Exos (70–150 nm)	miR-223	Promoted the fibroblast to myofibroblast transition (EMT) by activating TGF-β pathway	^103^
Diabetic nephropathy	- RAW264.7 cell line with high glucose - Precipitation	Exos (40–100 nm)	Not studied		^108^
Lung fibrosis	- RAW264.7 cell line with SiO_2_ - Ultracentrifugation	Exos (30–150 nm)	miR-125a-5p		^105^
	- Rat primary peritonea macrophages with IL-4 - Precipitation	Exos (undefined)	miR-328		^106^
	- THP-1 cell line with PMA and asbestos - Not given	Exos (undefined)	lncRNA-ASLNCS5088		^107^
	- THP-1 cell line with PMA and asbestos - Precipitation	Exos (undefined)	Not studied	Up-regulated genes *hCCNB2*, *hEGR1*, and *hFANCD2;* down-regulated genes *hCRELD2*, *hERO1B,* and *hJAG1* in mesothelial cells	^104^
Diabetes cardiac dysfunction	- RAW264.7 cell line with high glucose and BMDM from diabetes mice - Precipitation	Exos (undefined)	HuR protein	Increased inflammatory response and fibrogenesis in cardiac fibroblasts	^109^

### Cardiovascular diseases

Cardiovascular diseases (CVDs), such as coronary heart disease, peripheral arterial disease, and cerebrovascular disease, are a class of disorders of heart and blood vessels. Macrophages have been recognized as vital players that participated in the most stages of CVDs. For example, in atherosclerosis, macrophages within plaques scavenge retained lipoproteins and transform into cholesterol-laden foam cells, which subsequently exacerbate inflammatory responses and accelerate atherosclerosis progression^67,68^. Although the detailed mechanisms remain elusively, recent studies have realized the important roles of Mφ-EVs in the development of CVDs. For example, EVs from atherogenic macrophages promote plaque formation in atherosclerosis. These EVs restrained the migration of naïve macrophage out of plaques and reduced the growth and tube formation of endothelial cells (ECs), increasing plague progression^69,70^. Moreover, excessive lipoproteins accumulation induces the release of Mφ-EVs enriched with lncRNA GAS5, triggering apoptosis in ECs^71^. Vascular smooth muscle cells (VSMCs) usually reside close to macrophage infiltration sites during vascular lesions^72^. Recently, EVs from M1-like macrophages have been proven to activate extracellular regulated protein kinase (ERK) and protein kinase B (Akt) pathway by transferring integrins, which in turn stimulate extracellular matrix (ECM) production and cell migration and adhesion in VSMCs, aggravating atherosclerosis^73^. Additionally, EVs from macrophages exposed to nicotine also induce VSMC proliferation and migration by delivering miR-21-3p to activate phosphatase and tension homolog (PTEN) in these cells^74^.

Cardiac remodeling is a subsequent pathological condition following severe CVDs. Although the detailed mechanism is still under investigation, macrophage infiltration has been regarded as one of the vital factors in this course^75,76^. Macrophage-derived EVs are taken up by cardiac fibroblasts, and a macrophage-fibroblast crosstalk has been proposed. After myocardial infarction (MI), EVs derived from proinflammatory macrophages deliver miR-155 into cardiac fibroblasts, inhibiting their proliferation, causing abnormal cardiac remodeling and increasing the incidence of cardiac rupture^77^. Furthermore, EVs isolated from macrophages under hypertension were enriched with miR-17 and induce the expression of cytokines, such as intercellular adhesion molecule-1 (ICAM-1) and plasminogen activator inhibitor-1 (PAI-1), in ECs via activating the NF-κB pathway, which ultimately promote cardiac inflammation and pathological cardiovascular remodeling^78^. During intracranial aneurysm (IA) development, Mφ-EVs transfer miR-155-5p to promote the proliferation and migration of smooth muscle cells by downregulating GREM1, a member of the antagonists of secreted bone morphogenetic protein^79^. Collectively, although the relevant mechanisms are still under debate, the considerable effects of Mφ-EVs during CVDs are indispensable to disease development.

### Metabolic diseases

Metabolic diseases, such as obesity, insulin resistance (IR), and diabetes, belong to a type of complicated metabolic disorders with both high morbidity and mortality^80,81^. Macrophages-mediated inflammatory responses are recognized as an essential pathological process to disease progression. For example, increased numbers of adipose tissue macrophages (ATMs) have been observed in IR individuals^82^ and the morbidity of obesity and IR strongly parallels with the severity of inflammation^83^. Moreover, co-culture of ATMs with pancreatic cells demonstrated that cell–cell communication is responsible for inflammatory milieu formation^84^. EVs from obese ATMs induce glucose intolerance and IR, while these adverse effects could be reversed by EVs from lean ATMs^85^. PPARγ is a key factor for maintaining insulin sensitivity, and EVs from proinflammatory macrophages induce glucose intolerance and IR by disrupting PPARγ signaling. EVs isolated from obesity mice-derived ATMs deliver miR-155 and miR-29a to inhibit PPARγ in adipocytes, myocytes, and hepatocytes, increasing levels of fasting blood glucose, serum insulin, and index of homeostasis model assessment of IR in lean mice^85,86^. However, another study reported that EVs from LPS-induced macrophages failed to influence glucose uptake and fat storage in adipocytes but were able to up-regulate inflammation and carbohydrate catabolism related genes such as CXCL5, SOD, C3, and CD34 in adipocytes^87^. Additionally, macrophages derived from mice with knockout of *Ercc1*, a highly conserved endonuclease complex required for lesion excision in nucleotide excision repair, release EVs to induce glucose transporter type-1 (GLUT-1) expression in pancreatic cells, further leading to hyperglycemia and inflammation in primary pancreatic cells^88^. Overall, these findings collectively verified that Mφ-EVs promote metabolic diseases development by modulating glucose and lipid metabolism pathways.

### Infectious diseases

The key roles of macrophages against foreign invaders have been known for years. Investigators have recently realized that Mφ-EVs also exert several adverse roles during severe infections. Mycobacterium tuberculosis *(M.tb*)-induced tuberculosis (TB) is characterized by a series of severe inflammation responses along with the formation of granuloma and drug-resistance^89^. Several studies suggested that EVs from *M.tb* infected macrophages contribute to TB development. *M.tb* infected macrophages package mycobacterial lipids and proteins within their EVs, and these EVs further induce the formation of granuloma and facilitate *M.tb* spread^90,91^. Furthermore, Mφ-EVs are also implicated in viral infections such as human immunodeficiency virus (HIV) and hepatitis C virus (HCV). An increasing number of EVs has been found in HIV-infected monocyte-derived macrophages (MDM). Despite these EVs differing in size, morphology, and content (miRNA and proteomic profiles), they were able to deliver viral constituents to the uninfected MDM and thus induce infection in these cells^92,93^. Epidemiological data indicate that individuals infected with HIV are at high risk for the development of pulmonary diseases, but the underlying mechanism whereby this occurs remains unclear^94^. Interestingly, HIV-infected macrophages were found to release EVs plentiful in miR-130a, which induces pulmonary smooth muscle cell proliferation and development of pulmonary arterial hypertension^92^. Moreover, EVs from HIV-infected macrophages are rich in miR-23a and miR-27a, which damaged the EC integrity and caused mitochondrial dysfunction, ultimately exacerbating pulmonary lesions^93^. These findings suggest that EVs from infected macrophages can transfer the pathogenic constitutes or miRNAs to aggravate pathogen spread or disease severity. However, the ability of EVs to carry pathogenic constituents may also shed light on their application for pathogen vaccines.

### Tumor development and chemoresistance

Throughout the tumor microenvironment (TME), multiple cell type such as immune cells, vascular endothelial cells, and fibroblasts collectively work to regulate tumorigenesis. Disorders of the TME increase the occurrence of tumor recurrence, metastasis, and chemoresistance^95^. TAMs are one of the major TME cells that provide an immunosuppressive milieu for tumor progression and secrete many growth factors that suppress the proinflammatory cytokine release in the TME. It is thought that TAM-derived EVs (TAM-EVs) exhibit similar tumor-friendly properties to their donor cells. These EVs have shown the capacity to mediate cell-to-cell communication between TAMs and other immune cells such as T cells. For example, TAM-EVs enriched with miR-21-5p and miR-29a-3p inhibit the STAT3 pathway and induce Treg/Th17 cell imbalance, generating an immunosuppressive milieu to facilitate the progression and metastasis of epithelial ovarian cancer^96^. TAM-EVs also deliver miR-501 to activate the TGF-β pathway and promote tumor migration and invasion in pancreatic ductal adenocarcinoma^97^. Moreover, TAM-EVs play important roles in the pathology of tumor chemoresistance. It has been reported that EVs from TAMs under hypoxic conditions with enriched miR-223 promote drug resistance in epithelial ovarian cancer through activating PTEN-PI3K/AKT pathways^98^. However, another study argued that EVs derived from TAMs display molecular profiles associated with Th1/M1 polarization, and they might exhibit antitumor immunity^99^. Taken together, TAM-EVs have emerged as vital mediators in tumors development and chemoresistance, and their detailed effects in tumors still need to be investigated in future studies.

### Tissue fibrosis

Tissue fibrosis is characterized as the up-regulation of profibrotic factors (e.g., TGF-β, CTGF) and ECM components (e.g., Fibronectin, Collagen type I) that destroys normal histological structures, ultimately leading to organ dysfunction^100,101^. Recently, Mφ-EVs have been implicated in the pathology of tissue fibrosis, as their administration restarts the profibrotic pathways after depletion of macrophages in mice^102^. Asbestos exposure is a major cause of several severe lung diseases such as malignant mesothelioma (MM), lung fibrosis (asbestosis), and bronchial carcinoma. Evidence has shown that EVs from asbestos exposed macrophages induce the expression of many genes that are involved in TGF-β-meditated cell-cycle control and chromosome instability in mesothelial cells, contributing to the development of lung fibrosis^103^. Lung silicosis induced by silica particles is primarily characterized by diffuse fibrosis. EVs from macrophages exposed to silica also up-regulate α-SMA expression in fibroblasts due to the abundant miR-125a-5p in these EVs^104^. Moreover, EVs from M2-like macrophages are enriched with miR-328 and lncRNA-ASLNCS5088, which subsequently activates TGF-β and profibrotic factors (e.g., α-SMA, Collagen I) in fibroblasts^105,106^.

Progressive renal fibrosis is a hallmark of diabetic nephropathy (DN), a leading cause of end-stage renal disease (ESRD) and renal failure in huge number of diabetic patients. Although macrophage infiltration is linked to the progression of DN, their roles in renal fibrosis during DN are not entirely clear. Interestingly, a recent study found that EVs from macrophages under high glucose condition were taken up by mesangial cells, enhancing the proliferation and excessive ECM secretion in these cells via activating TGF-β1/Smad3 pathways^107^. Mφ-EVs from diabetic conditions were abundant with HuR, an RNA-binding protein that regulates post-transcription, and induce fibrogenesis in fibroblasts^108^. As a result, angiotensin II-induced CVD mice supplemented with EVs carrying HuR displayed clear interstitial and perivascular fibrosis^108^. Collectively, these findings have revealed the significant roles of EVs in the pathology of tissue fibrosis in multiple organs.

## Therapeutic roles of Mφ-EVs in diseases

Depending on their parental cell types and the cargos they carry, Mφ-EVs may exhibit either immunosuppressive or immunostimulatory effects, thus serving as potential tools for disease therapy. As a type of endogenous nanovesicles, EVs have several advantages such as low immunogenicity, nontoxicity, and higher stability, over other synthetic nanoparticles^109^. Moreover, EVs exhibit the ability to pass through tissue barriers, such as the BBB^110^, enhancing the therapeutic efficacy of incorporated molecules to target cells. Mφ-EVs have emerged as a promising cell-free therapy for many biomedical applications such as tissue repair, drug delivery, and pathogen control (Table 2 and Figs. 3 and 4).

**Table 2:** Summary of the therapeutic roles of Mφ-EVs in diseases.

Disease models	EVs source and Isolation method	EV subtypes (diameter)	Therapeutic molecules	EV doses and routes	Therapeutic outcomes	References
*Tissue repair*						
Myocardial I/R injury	- Rat peritonea macrophages - Ultracentrifugation	Exos (~100 nm)	miR-148a	- 2 h before I/R procedure - 2–3 μg per rat by single caudal vein	- Reduced the dysregulation of cardiac enzymes and Ca^2+^ overload - Reduced apoptosis and the number of broken cardiomyocytes	^112^
Diabetic skin defects	- RAW 264.7 cell line - Ultracentrifugation	Exos (~95 nm)	Not studied	- The day when wounds were produced - 0.1 or 1 mg EVs in 1 ml PBS per rat by single subcutaneously	- Elevated angiogenesis, migration and proliferation ability of high glucose treated HUVECs by anti-inflammation - Accelerated wound contraction and reduced wound length - Therapeutic effects with dose-dependent	^113^
Hair loss	- RAW 264.7 cell line - Ultracentrifugation	EVs (~128.8 nm)	Wnt3a/Wnt7b	- 2 days after the hair removed - 0.1 or 1 mg EVs per mouse by intradermally three times weekly for 4 weeks	- Increased proliferation and migration of dermal papilla cells by activating Wnt/β-Catenin pathway - Increased hair follicle number, elongation of hair and thickness of dermis with time- and dose-dependent - Therapeutic effects with dose-dependent	^114^
Radiation-induced gastrointestinal syndrome	- Mice BMDM - Ultracentrifugation and precipitation	EVs (undefined)	Functional Wnt ligands	Unclear	Elevated survival of mice with wild type BMDM cell medium	^115^
Inflammatory bowel disease	- Mice BMDM with IL-13/IL-10/IL-1β - Precipitation	Exos (30–150 nm)	CCL1	- The day when administration with DSS - 50 mg/mouse by intraperitoneally once a day for 8 days	- Reduced length and inflammatory damage of colon - Increased number of Treg cells in spleen - Alleviated weight loss, diarrhea and bleeding in mice with colitis	^116^
Cutaneous Wound	- Mice BMDM - Ultracentrifugation	Exos (~69 nm)	not specific	- 100 μg/mouse by subcutaneously injection - Once a day at day 1 and 4	- Increased expression of Arginase and decreased expression of iNOS in M1-like Mφ - Enhanced fibroblast migration and EC tube formation - Increased wound dermal cellularity and collagen production	^117^
*Pathogen control*						
Systemic candidiasis	- THP-1 cell line with Candida albican - Ultracentrifugation	EVs (~30–369 nm)	Proteins	Unclear	- Elevated proinflammatory cytokines by activating ERK2 and p38 - Decreased candidacidal activity	^122^
Tuberculosis	- THP-1 cell line with *mycobacteria* - Sucrose gradient centrifugation	Exos (50–100 nm)	Not studied	- 20 μg EVs per mouse by intranasally - Single dose	- Elevated inflammatory response	^118^
	- Mouse BMDM with *M.tb* - Ultracentrifugation	EVs (undefined)	*M.tb*-RNA	- 3 weeks after infection - 5 μg/mouse EVs intratracheally at 4 weeks post‐infection	- Decreased replication and survival of *M.tb* by increased IFN-γ - Elevated autophagy by activating LC-3-associated phagocytosis pathway	^119^
	- RAW 264.7 cell line with *M.tb* culture filtrate protein - Ultracentrifugation	Exos (50–150 nm)[112] Exos (30–100 nm)[113]	Immunized purified EVs	- Six weeks before *M.tb* infection - 40 μg/mouse EVs three times by intranasally at an interval of 2 weeks	- Elevated Th1 response and limited Th2 response - Reduced mycobacterial numbers in lung	^120,121^
Dengue	- U937 Mφ with DENV - Ultracentrifugation	EVs (~100 nm)	NS3 and miRs	Unclear	Elevated production of proinflammatory cytokines in ECs	^123^
HCV	- Human monocytes-derived Mφ with HIV RNA - Precipitation	Exos (50–100 nm)	miR-29	Unclear	Decreased HCV replication by inducing the expression of antiviral genes such as IFN-α, ISGs, OAS-1	^124^
	- THP-1 cell line with IFN - Ultracentrifugation	EVs (50–400 nm)	Not studied	Unclear	Profound inhibition of HCV RNA replication after 72 h or 96 h exposure	^126^
*Drug delivery*						
Pulmonary metastases	- RAW 264.7 cell line for vitro; mice BMDM for vivo - Precipitation	Exos (~110 nm)	EVs loaded with PTX	- 40 h after i.v. injecting tumor cells - 4 × 10^11^ particles per mouse by i.v. at the days 1, 4, and 7	- Higher drug loading and targeting capacity towards cancer cells - Superior antineoplastic efficacy and prolonged lifespan	^132^
Breast cancer	- RAW 264.7 cell line - Ultracentrifugation	EVs (~110.8 nm)	EVs loaded with DOX or PTX	- Tumor volume reached ~50 mm^3^ - EVs-PTX (0.5 mg/kg); EVs-DOX (2.5 mg/kg) by i.v. once every 3 days	- Improved loading efficiency of DOX when pH close to PI, of PTX when dissolved in ethanol - Higher affinity towards tumor sites, more robust inhibitory potency on tumor growth and prolonged lifespan of both	^133^
	- RAW 264.7 cell line - Ultracentrifugation	Exos (~97.3 nm)	EVs loaded with DOX	- Tumor volumes reached 60 mm^3^ - At DOX dose of 5 mg/kg by i.v. every 3 days for total 18 days	- Prolonged circulation time of DOX and better accumulation of DOX at tumor tissue - Highest tumor inhibition efficacy - Decreased other tissue lesions	^134^
	- J774A.1 cell line - Membrane filter extrusion	EVs (~139 nm)	Hybrid EVs with liposomes and loaded with DOX	Unclear	- Higher drug release in acidic microenvironment - Higher biocompatibility as a safe delivery system with lower cytotoxicity - Decreased K7M2, 4T1, and NIH/3T3 cell viability	^135^
*Vaccine adjuvant*						
Melanoma	- RAW264.7 cell line with γ-IFN - Ultracentrifugation	Exos (~50 nm)	cytokines such as IL-6, IL-12, and γ-IFN	- Tumor volume reached ~50 mm^3^ - 10 μg EVs by single subcutaneously at 24 h after injection of vaccine	- Elevated apoptosis of melanoma cancer cells - Decreased celluar scar tissues as well as many infiltrating immune cells - Significant inhibitory effects on tumor growth	^131^

### Tissue repair

Functional recovery of injured tissues remains a huge clinical challenge, and Mφ-EVs have shown several beneficial effects on injured cells or tissues. For example, myocardial ischemia/reperfusion (I/R) injury is characterized by irreversible injury to the myocardium and results in heart dysfunction. Interestingly, EVs from M2-like macrophages deliver miR-148a to mitigate I/R-induced myocardial injury via suppressing the overloaded Ca^2+^ and inflammatory cytokine production in cardiomyocytes^111^. The anti-inflammatory effect of Mφ-EVs was also investigated in the models of diabetic wound, which is characterized by a persistent inflammatory response. For example, the skin defect in diabetic rats was rescued by EVs derived from anti-inflammatory macrophages^112^. Mechanistically, these EVs not only reduced cytokine (e.g., TNF-α, IL-6) secretion in ECs, but also induced EC proliferation and migration to improve angiogenesis and re-epithelialization during wound healing^112^. Furthermore, Mφ-EVs promote hair growth and protect the intestine against radiation injury through activation of WNT/β-catenin signaling^113,114^.

Mφ-EV-guided immune cell reprogramming is a promising therapeutic approach for inflammation-associated disorders. EVs from M2b macrophages display a more robust protective capacity compared to those from M2a macrophages or M2c macrophages in dextran sulfate sodium-induced colitis. The anti-inflammatory effects of M2b-derived EVs may be ascribed to elevated CCL1, which promotes Th2 polarization of the colon to rebalance the Th1 immune response, thereby attenuating the severity of DSS-induced colitis in mice^115^. Mφ-EVs also induce phenotype switching in macrophages, and subcutaneous injection of M2-like macrophages-derived EVs in a cutaneous wound induced local macrophage phenotype switching from M1-like towards M2-like, promoting the cutaneous wound healing by enhancing angiogenesis, re-epithelialization, and collagen deposition^116^.

### Pathogen control

Macrophages are one type of primary innate immune cells that can fight pathogen infection. The therapeutic role of Mφ-EVs also sheds light on infectious diseases. For instance, EVs from *M.tb*-infected macrophages are capable of inducing the systematic inflammatory responses with high levels of cytokines (e.g., TNF-α, IL-12) in primary macrophages and *M.tb*-infected mice, which was indispensable at the early phase of pathogen control^91,117^. Similarly, EVs derived from *M.tb*-infected macrophages transfer *M.tb* RNA into naïve macrophages and activate the intracellular RNA sensing pathway, promoting Th1 immune response with the release of IFNs^118^. In addition, Mφ-EVs carry viral proteins to irritate the immune system during infections. It has been found that EVs from *M.tb*-infected macrophages carried mycobacterial proteins, and these EV-vaccinated mice exhibited a robust Th1 response with limited Th2 response, suggesting that Mφ-EVs may be regarded as promising cell-free agents against TB^119,120^. During the early stage of fungal infection, EV contents from *Candida albicans*-infected macrophages were extensively altered and were intimately associated with enhanced cytokine secretion in macrophages^121^. Moreover, EVs from dengue virus (DENV)-infected macrophages also transfer the non-structural protein (NS3) encoded by DENV RNA to promote cytokine release in ECs, activating the defense program against dengue virus infection at the early stage^122^. EVs released from toll-like receptor 3 (TLR3)-activated macrophages, which are often blocked during infection, were found to possess abundant miR-29 to inhibit HCV replication in hepatocytes^123^. Interestingly, it seems that macrophages may have certain mechanisms to mediate the release pattern and synergistic effects of cytokines and EVs in response to infections. For example, macrophages orchestrate a fast but short-lasting antiviral state by secreting cytokines during HCV infection, while their EVs induce a late but long-lasting inhibition on HCV replication in hepatocytes with sub-genomic HCV replicons^124^. These studies suggest that Mφ-EVs may boost the anti-infective immune response by delivering viral materials or therapeutic molecules.

### Drug delivery

EVs have lipid bilayer membrane structures, allowing them to encapsulate and deliver various types of bioactive molecules in vivo^36,125^. The nanoscale size and natural bilipid membrane of EVs allow them to easily pass through biological barriers (e.g., blood–brain barrier)^126,127^. Moreover, EVs from macrophages express CD47, a surface molecule known as the “don’t eat me” signal to escape immunological surveillance^128,129^. Therefore, application of Mφ-EVs as drug delivery vehicles has received considerable attraction. EVs from M1-like macrophages alone showed a mild antitumor effect^97,130^, but they were capable of restricting tumor growth when combined with other therapeutic agents. For example, Mφ-EVs loaded with Paclitaxel (PTX) exhibited a robust accumulation in Lewis lung carcinoma (LLC) cells both in vitro and in vivo. The EV-PTX combination significantly reduced lung metastases and prolonged lifespan in an LLC metastasis mouse model^131^. Although Mφ-EVs have shown potential for cancer therapy, their poor drug loading efficacy largely impedes further clinical translation. Thus, optimized drug loading processes for Mφ-EVs have also been studied. For example, the loading efficiency of Mφ-EVs with Doxorubicin (Dox) was improved when pH was close to the pI of Dox, and PTX dissolved in ethanol was efficiently encapsulated into Mφ-EVs. Accordingly, these optimized drug-loaded Mφ-EVs displayed higher inhibitory potency on tumor growth in orthotopic triple-negative breast cancer (TNBC) tumor models compared to the original drug-loaded EVs or drug alone^132^.

In addition, some hybrid strategies have been proposed to enhance the therapeutic potency of Mφ-EVs. For example, TNBC is aggressive and often returns after treatment. To resolve this issue, Mφ-EVs were engineered by removing their own contents and modifying addition of c-Met onto their surface, after which Dox-loaded poly lactic-co-glycolic acid (PLGA) nanoparticles were wrapped into the empty Mφ-EVs. These engineered EVs prolonged the release profile of Dox in vitro, and displayed high tumor targeting ability and excellent tumor inhibitory efficiency in mice with TNBC^133^. Another example is hybridizing EVs from M1-like macrophages with liposomes: the resulting hybrid Mφ-EVs exhibited higher cytotoxicity to multiple types of cancer cells, such as osteosarcoma and breast cancer cells, when loaded with Dox^134^. These studies indicate that Mφ-EVs constitute a promising natural carrier for target drug delivery. However, despite these efforts, obstacles are still substantively unresolved with respect to obtaining EVs with the desires properties.

### Adjuvant cancer vaccines

Cancer immunotherapy functions are primarily based on the patient’s own immune system, and it is believed to be an important shift in tumor therapy. Cancer vaccines are commonly needed in combination with other therapies or adjuvants to improve the magnitude, breadth, quality, and longevity of the immune response to vaccines^135^. Although great progress has been made recent years, some limitations still exist in many adjuvants, such as induction of disabled immune responses. Recently, EVs derived from the proinflammatory macrophages have been proposed as promising adjuvants. Exogenous supplementation with EVs from M1-like macrophages skewed naïve macrophages towards a proinflammatory state within 4 h, generating a proinflammatory microenvironment that favored Th1 immune response. However, this dramatic effect of EVs was transient and gradually faded^130^. Hence, optimum treatment time must be taken into consideration. Cheng et al. employed a peptide vaccine in combination with M1-like EVs in a melanoma model. These EVs were given 4 h before the peptide vaccine arrived at the tumor, and this combination therapy exhibited stronger antitumor effects compared to traditional adjuvants such as TLR agonist^130^. Mechanically, M1-like EVs might serve as an agent to stimulate proinflammatory milieu, which makes the immune system more sensitive to cancer vaccines, thereby inducing a superior antitumor effect. These findings strongly indicate that EVs from M1-like macrophages are a promising immune adjuvant suitable for cancer vaccines.

## Conclusions and future perspectives

Notably, macrophages are key mediators in the innate immune system, and are involved in the pathology of many diseases. EVs are capable of exerting several functions similar to their parent cells, and have received increasing attention due to their natural portability and extraordinary actions on target cells. Similar to their parent cells, Mφ-EVs function extensively in the pathology of various diseases, as well as being robust mediators for immunotherapy, serving as therapeutics for many diseases. Employment of Mφ-EVs has several advantages. First, Mφ-EVs can easily escape immunological surveillance due to abundant immune molecules such as CD47 on their surface, which help them escape immune attack^129^. Second, the ability of Mφ-EVs to either enhance or suppress immune activity makes them attractive candidates for various diseases as discussed in this review. Third, administration of EVs as a substitute for Mφ might reduce some of the risks associated with whole cell therapy such as cytokine release syndrome, also known as a cytokine storm^136^. Moreover, EVs are nanoscale and circulate readily compared to larger Mφ, and they can be further modified and used as potential drug delivery systems due to their ability to cross biological barriers^137^.

However, several limitations or issues raised by Mφ-EVs should be taken into consideration before future clinical translation. After recognition of the pathogenicity of Mφ-EVs in diseases, it is crucial to further investigate how to regulate their release or their embedded contents properly to prevent development and progression of disease. Additionally, macrophages are able to exert immediate and diverse responses to different microenvironments, and this unique property seems important to their robust therapeutic effects in multiple diseases. However, unlike whole cells, Mφ-EVs alone are not believed to have similar action, and thus precise modulation of Mφ-EV properties should be conducted in response to different diseases or conditions before intervention. Reprogramming or reengineering of Mφ-EVs for disease therapy seems promising for the future, while the poor yield and/or limited function of Mφ-EVs using traditional methods has largely impaired their further biomedical applications, and thus new isolation or manufacture methods with higher yield/function of EVs are urgently needed. Nevertheless, these problems are not exclusively confined to macrophages and answers may come from academic studies devoted to the biological properties of EVs. Overall, the potential of macrophage-derived EVs in immunoregulation and disease intervention is highly promising.
